# Self- versus caregiver-reported apathy across neurological disorders

**DOI:** 10.1093/braincomms/fcaf235

**Published:** 2025-06-13

**Authors:** Sijia Zhao, Anna Scholcz, Matthew A Rouse, Verena S Klar, Akke Ganse-Dumrath, Sofia Toniolo, M John Broulidakis, Matthew A Lambon Ralph, James B Rowe, Peter Garrard, Sian Thompson, Sarosh R Irani, Sanjay G Manohar, Masud Husain

**Affiliations:** Department of Experimental Psychology, University of Oxford, Oxford OX2 6GG, UK; Department of Experimental Psychology, University of Oxford, Oxford OX2 6GG, UK; Cognitive Disorders Clinic, John Radcliffe Hospital, Oxford OX3 9DU, UK; MRC Cognition and Brain Sciences Unit, University of Cambridge, Cambridge CB2 7EF, UK; Department of Experimental Psychology, University of Oxford, Oxford OX2 6GG, UK; Department of Experimental Psychology, University of Oxford, Oxford OX2 6GG, UK; Cognitive Disorders Clinic, John Radcliffe Hospital, Oxford OX3 9DU, UK; Cognitive Disorders Clinic, John Radcliffe Hospital, Oxford OX3 9DU, UK; Nuffield Department of Clinical Neurosciences, University of Oxford, Oxford OX3 9DU, UK; Department of Experimental Psychology, University of Oxford, Oxford OX2 6GG, UK; Cognitive Disorders Clinic, John Radcliffe Hospital, Oxford OX3 9DU, UK; MRC Cognition and Brain Sciences Unit, University of Cambridge, Cambridge CB2 7EF, UK; MRC Cognition and Brain Sciences Unit, University of Cambridge, Cambridge CB2 7EF, UK; Department of Clinical Neurosciences, University of Cambridge, Cambridge CB2 0QQ, UK; Cambridge University Hospitals NHS Foundation Trust, Cambridge CB2 0QQ, UK; Neuroscience and Cell Biology Research Institute, City St George’s, University of London, London SW17 0RE, UK; Cognitive Disorders Clinic, John Radcliffe Hospital, Oxford OX3 9DU, UK; Nuffield Department of Clinical Neurosciences, University of Oxford, Oxford OX3 9DU, UK; Department of Neurology, Mayo Clinic, Jacksonville, FL 32224, USA; Department of Neurosciences, Mayo Clinic, Jacksonville, FL 32224, USA; Department of Experimental Psychology, University of Oxford, Oxford OX2 6GG, UK; Cognitive Disorders Clinic, John Radcliffe Hospital, Oxford OX3 9DU, UK; Nuffield Department of Clinical Neurosciences, University of Oxford, Oxford OX3 9DU, UK; Department of Experimental Psychology, University of Oxford, Oxford OX2 6GG, UK; Cognitive Disorders Clinic, John Radcliffe Hospital, Oxford OX3 9DU, UK; Nuffield Department of Clinical Neurosciences, University of Oxford, Oxford OX3 9DU, UK

**Keywords:** rating discrepancy, informant report, dementia, impaired insight, cognitive impairment

## Abstract

Apathy is a prevalent and persistent neuropsychiatric syndrome across many neurological disorders, significantly impacting both patients and caregivers. We systematically quantified discrepancies between self- and caregiver-reported apathy in 335 patients with a variety of diagnoses, such as frontotemporal dementia (behavioural variant and semantic dementia subtypes), Parkinson’s disease, Parkinson’s disease dementia, dementia with Lewy bodies, Alzheimer’s disease dementia, mild cognitive impairment, small vessel cerebrovascular disease, subjective cognitive decline and autoimmune encephalitis. Using the Apathy Motivation Index (AMI) and its analogous caregiver version (AMI-CG), we found that caregiver-reported apathy consistently exceeded self-reported levels across all conditions. Moreover, self-reported apathy accounted for only 14.1% of the variance in caregiver ratings. This apathy reporting discrepancy was most pronounced in conditions associated with impaired insight, such as behavioural variant frontotemporal dementia, and was significantly correlated with cognitive impairment. Deficits in memory and fluency explained an additional 11.2% of the variance in caregiver-reported apathy. Specifically, executive function deficits (e.g. indexed by fluency) and memory impairments may contribute to behavioural inertia or recall of it. These findings highlight the need to integrate patient and caregiver perspectives in apathy assessments, especially for conditions with prominent cognitive impairment. To improve diagnostic accuracy and deepen our understanding of apathy across neurological disorders, we highlight the need for adapted apathy assessment strategies that account for cognitive impairment particularly in individuals with insight or memory deficits. Understanding the cognitive mechanisms underpinning discordant apathy reporting in dementia might help inform targeted clinical interventions and reduce caregiver burden.

## Teaser

Zhao *et al*. report that individuals with neurological conditions tend to underestimate their own apathy, particularly in disorders characterized by more severe cognitive impairment and lack of insight. The discrepancy between self- and caregiver-reported ratings highlights the need to integrate both perspectives when assessing motivation-related symptoms in clinical settings.

## Graphical Abstract

**Graphical Abstract:**
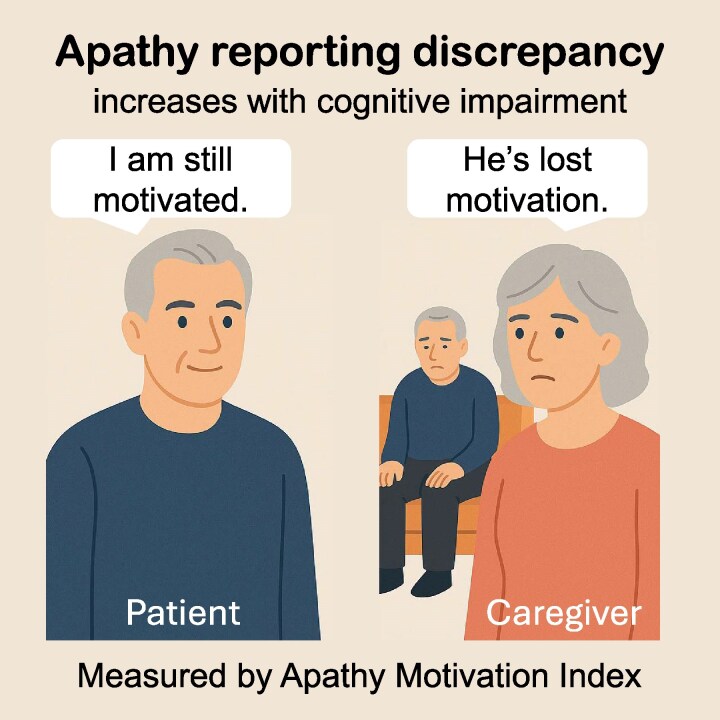

## Introduction

Apathy is defined as a syndrome of reduced goal-directed behaviours, including social activity or emotional responsiveness, associated with reduced motivation affecting and significantly impacting on everyday life.^1^ It is increasingly recognized as a prevalent and persistent problem across neurodegenerative disorders^2-7^ and associated with functional impairment, independent of disease severity and other covariates (e.g. age, sex and depression).^8-15^ The syndrome presents significant challenges for both patients and caregivers. Apathetic individuals often rely on caregivers to initiate activities they previously used to perform independently.^16^ Apathy may be associated with lower quality of life, although it may also protect against distress in the presence of impairments.^14,17-21^ At the same time, caregivers often misinterpret apathy as a voluntary choice by the patient not to engage, which makes it one of the most distressing neuropsychiatric symptoms they report.^14,17-21^ Accurate assessment of apathy is therefore crucial for effective management, but this can be challenging due to potential discrepancies between patient self-report and caregiver perspectives.^22,23^ However, while apathy is prevalent across all dementias, the extent to which self- or caregiver reports captures it differs between disorders, highlighting the need for a systematic evaluation of this discrepancy.

Previous research, using qualitative interviews^24-26^ or established questionnaires such as the Lille Apathy Rating Scale^27^ and Apathy Evaluation Scale,^28^ has examined the discrepancy in apathy between patients and caregivers in Alzheimer’s disease dementia (Ad), frontotemporal dementia (FTD) and Parkinson’s disease (PD).^27,29-31^ These studies revealed that caregivers generally perceived patients as more apathetic than the patients self-reported, and that the correlation between caregiver and patient assessments is low.^24-27,29-32^ This may reflect caregiver overestimation, potentially due to increased burden,^27,30^ patient underestimation^24-26^ or patient violations of the assumptions underlying the use of rating scales.^33^ Underestimations from self-reporting has been linked with cognitive impairment in dementia, which may represent anosognosia.^34-38^ Impaired awareness of one’s own deficits has been associated with greater apathy severity in Ad, without corresponding increases in anxiety or depression.^36,37,39,40^ A recent report showed that this phenomenon was particularly prominent in FTD.^40^

Here we used the Apathy Motivation Index (AMI), a validated self-report measure of apathy,^41^ and its caregiver-report counterpart (AMI-CG),^42^ to investigate the discrepancy between self- and caregiver-reported apathy across ten neurological conditions: behavioural variant FTD (bvFTD), semantic dementia (SD), PD, PD dementia (PDD), dementia with Lewy bodies (DLB), Ad, mild cognitive impairment (MCI), small vessel cerebrovascular disease (SVD), autoimmune encephalitis (AIE) and subjective cognitive decline (SCD). Apathy is extremely common in bvFTD,^15,32,43-45^ with most studies reporting greater than 70% patients affected at some point in their illness.^46^ In PDD and DLB, apathy also affects over 70% of individuals and is associated with increased caregiver burden and functional impairment.^47,48^ Here we systematically compare self- and caregiver-reported apathy across this range of conditions using analogous scales.

The present study systematically aimed to examine discrepancies between self- and caregiver-reported apathy—termed apathy reporting discrepancy (ARD)—across a broad range of neurological conditions using the same apathy measure (AMI). While previous studies have reported that informant-rated apathy tends to exceed patient self-ratings, it remains unclear whether this pattern generalizes across conditions or reflects disorder-specific mechanisms. We therefore hypothesized that (i) caregiver-reported apathy would consistently exceed self-reports across diagnostic groups and (ii) the magnitude of ARD would correlate with the severity of cognitive impairment. Confirmation of these hypotheses would highlight the need to educate caregivers on how cognitive dysfunction and specific diagnoses (e.g. bvFTD) influence on apathy awareness. This knowledge could help alleviate caregiver burden and facilitate the development of targeted support strategies.

## Materials and methods

### Ethics statement

The study was performed in accordance with the Declaration of Helsinki. All participants, including caregivers, gave written informed consent. Ethical approval was granted by the University of Oxford ethics committee (IRAS ID: 248379, Ethics Approval Reference: 18/SC/0448). For the Cambridge and London participants, ethical approval was granted by the National Research Ethics Service’s East of England Cambridge Central Committee (IRAS ID: 252986).

### Participants

This study included 335 patients with various neurological conditions and their respective caregivers. The patient cohort comprised the following 10 neurological conditions: bvFTD (*N* = 44), SD (*N* = 15), SVD (*N* = 24), PD (*N* = 58), PDD (*N* = 19), DLB (*N* = 18), Ad (*N* = 54), MCI (*N* = 7) and SCD (*N* = 43) (Table 1).

**Table 1:** Demographics and clinical characteristics of participants^a^

Diagnosis	*N*	Gender	Age	ACE total	AMI-SR total	AMI-CG total
bvFTD	43	F: 13, M: 30	62.95 (9.55, *N* = 40)	66.11 (15.82, *N* = 38)	1.42 (0.79, *N* = 43)	2.63 (0.74, *N* = 43)
SD	16	F: 11, M: 5	64.69 (7.43, *N* = 16)	59.81 (14.46, *N* = 16)	1.22 (0.35, *N* = 16)	1.69 (0.76, *N* = 16)
SVD	24	F: 4, M: 20	70.08 (7.56, *N* = 24)	80.50 (18.43, *N* = 22)	1.38 (0.50, *N* = 24)	1.77 (0.66, *N* = 24)
AIE	53	F: 18, M: 35	63.38 (13.45, *N* = 53)	90.85 (6.45, *N* = 47)	1.39 (0.57, *N* = 53)	1.56 (0.68, *N* = 53)
PD	58	F: 23, M: 35	68.47 (7.63, *N* = 58)	94.40 (3.53, *N* = 53)	1.41 (0.44, *N* = 58)	1.58 (0.60, *N* = 58)
PDD	19	F: 4, M: 15	71.05 (7.55, *N* = 19)	73.21 (11.97, *N* = 19)	1.60 (0.55, *N* = 19)	2.11 (0.66, *N* = 19)
DLB	18	F: 4, M: 13^c^	69.24 (7.23, *N* = 17)	75.61 (15.81, *N* = 18)	1.48 (0.62, *N* = 18)	2.03 (0.65, *N* = 18)
Ad	54	F: 24, M: 30	69.33 (9.39, *N* = 54)	67.82 (16.61, *N* = 51)	1.58 (0.56, *N* = 54)	1.86 (0.68, *N* = 54)
MCI	7	F: 6, M: 1	64.57 (6.90, *N* = 7)	85.29 (5.59, *N* = 7)	1.26 (0.22, *N* = 7)	1.72 (0.59, *N* = 7)
SCD	43	F: 20, M: 23	57.02 (9.98, *N* = 43)	91.88 (7.89, *N* = 43)	1.66 (0.48, *N* = 43)	1.63 (0.68, *N* = 43)
HC	19	F: 11, M: 8	64.26 (6.71, *N* = 19)	97.16 (1.57, *N* = 19)	1.12 (0.31, *N* = 19)	0.92 (0.46, *N* = 19)
All patients^b^	335	F: 127, M: 207	65.68 (10.39, *N* = 331)	80.48 (17.04, *N* = 314)	1.47 (0.56, *N* = 335)	1.84 (0.75, *N* = 335)

^a^For continuous variables (age, length of patient-caregiver relationship, etc.), the mean and standard deviation (SD) are reported, along with the number of participants for whom this information was available (indicated in parentheses).

^b^‘All patients’ refers to all diagnostic groups combined, excluding HCs.

^c^One patient's gender information was missing.

The majority of patients (*N* = 247) were recruited from the Cognitive Disorders Clinic at John Radcliffe Hospital, Oxford, UK. All AIE patients (*N* = 53) were recruited from Autoimmune Neurology Clinic. From specialist dementia clinics in Addenbrooke’s Hospital, Cambridge, UK (*N* = 31) and St George’s Hospital, London, UK (*N* = 4), 35 patients (all with FTD, either bvFTD or SD) were recruited. Nineteen older healthy controls (HCs) and their partners were also included, with most of these dyads (18/19) recruited in Cambridge. The questionnaire data were collected between 12 July 2016 and 6 May 2024. All diagnoses were made by experienced neurologists according to consensus clinical diagnostic criteria.

Demographic information, including patient and caregiver age, gender, relationship to the patient, and length of the caregiver–patient relationship, was collected for most participants. Demographic characteristics for each diagnostic cohort are summarized in Table 1 (see Supplementary Table 1 for more details).

Caregiver relationship to the patient was recorded for 298 of the Oxford patients. Of these, 237 were spouses or partners, 29 were children, 21 were siblings or other family members, and 11 were friends. No professional caregivers were included in this sample. The majority of caregivers were female (79.6% of 137 caregivers with gender recorded).

To be included in the study, caregivers were required to know the patient well, defined as either being a spouse/partner (79.5% of the sample) or having known the patient for at least 3 years. On average, caregivers had known the patients for 39.2 years (SD = 15.4 years; see Supplementary Table 1 for cohort-wise statistics).

### Measures

Apathy: All patients completed the AMI Self-reported version (here referred as AMI-SR),^41^ and their caregivers completed the caregiver version (AMI-CG).^42^

- AMI-SR: This 18-item self-report questionnaire assesses apathy across three domains: Behavioural Activation, Social Motivation, and Emotional Sensitivity. Participants respond using a 5-point Likert scale. Item scores are averaged to yield subscale and total scores, with higher scores indicating greater apathy (range 0–4). A cut-off score of ≥1.91 (1 SD above the mean of a healthy population) was used to identify apathy.^41^
- AMI-CG: This measure uses the same items and response options as the AMI, with wording adapted to reflect a third-person perspective.^42^ Developed from the original AMI, this measure covers the same three domains of apathy: behavioural, social and emotional apathy. It is scored in the same way, with higher scores indicating greater apathy (range 0–4). Since the AMI-CG is adapted from the self-reported AMI questionnaire and contains identical item content, but no caregiver-specific cut-off score has been established, we applied the same cut-off (≥1.91) as used for AMI-SR.

ARD was defined as the difference between caregiver- and self-reported apathy, calculated by subtracting AMI-SR scores from AMI-CG scores.

Depression and anhedonia: Patients recruited in Oxford (*N* = 300) also completed the following:

- Snaith-Hamilton Pleasure Scale (SHAPS)^49^: This 14-item self-report questionnaire aims to assess hedonic tone across four domains: interest/pastimes, social interaction, sensory experience and food/drink. A 4-point response scale was used, with higher scores indicating greater anhedonia. Following Franken *et al*.,^50^ a 4-point scoring system was used to increase data dispersion for correlation analyses. A cut-off score of ≥22.3, derived from a recent meta-analysis, was used to identify clinically significant anhedonia.^51^ Data were available for 298 patients.
- Geriatric Depression Scale Short Form (GDS)^52^: This 15-item self-report screening tool aims to assess depressive symptoms in older adults excluding somatic symptoms, providing a more robust measure in individuals with neurodegenerative illnesses. A 2-point response scale (yes/no) was used, with scores ranging from 0 to 15; higher scores indicate more severe depression. An established cut-off score of ≥5 was used to identify possible depression.^53,54^ To minimize overlap with apathy, a GDS Depression subscale score was calculated, excluding the three *prima facie* apathy-related items: ‘Have you dropped many of your activities and interests?’, ‘Do you prefer to stay at home, rather than going out and doing new things?’, and ‘Do you feel full of energy?’. This adjustment did not affect the overall conclusions of the study. Data were available for 294 patients.

Cognitive function: Patients recruited in Oxford also completed the Addenbrooke’s Cognitive Examination-III (ACE-III)^55^ in person (*N* = 279), and 35 patients recruited in Cambridge completed the ACE-Revised (ACE-R).^56^ They are very similar and both assessed cognitive function across five domains: attention, memory, verbal fluency, language, and visuospatial skills. Scores range from 0 to 100, with lower scores indicating greater cognitive impairment. Both ACE-III and ACE-R scores >88/100 are generally considered normal.^57^ The total score (ACE Total) and individual subscores for each domain (ACE attention, ACE memory, ACE fluency, ACE language and ACE visuospatial) were reported here.

### Statistical analysis

All analyses and data visualization were performed using MATLAB (version R2024b), R statistical software (version 4.3.3)^58^ and R-based statistical software JASP.^59^

Exploratory factor analysis (EFA) was conducted using R package psych.^60^ The Kaiser–Meyer–Olkin measure of sampling adequacy was first computed to assess whether the sample was appropriate for factor analysis. To determine the optimal number of factors, Horn’s Parallel Analysis was conducted with 2000 iterations. EFA was then conducted using Promax rotation, which allows for inter-factor correlations. This choice was based on prior knowledge that the different subdomains of apathy measured by the AMI are interrelated. While this approach reduces factor orthogonality, we found no difference in factor structure when using Varimax rotation. Therefore, we chose to report the EFA results using Promax rotation. Reliability of the AMI-CG was assessed using Cronbach’s alpha, computed in JASP. Model fit was assessed using the Bayesian information criterion (BIC), root mean square error of approximation (RMSEA), standardised root mean squared residual (SRMR), Tucker-Lewis index (TLI), and comparative fit index (CFI).

All bivariate and partial correlations were performed using Spearman’s rank correlation. Following Cohen (1988),^61^ correlation coefficients ≥0.1, ≥0.3 and ≥0.5 were interpreted as representing weak, moderate and strong relationships, respectively. Differences in Spearman correlation coefficients were tested using the procedures for testing statistical differences between correlations using the implementation in the R package cocor.^62^ Fisher’s *z* was reported along with *P*-value (two-tailed).

All *P*-values reported are two-tailed. Group comparisons were performed using paired *t*-tests for paired data. For between-group comparisons with unequal sample sizes, continuous variables were compared using the Mann–Whitney U-test, and categorical variables were compared using the *χ*^2^ test. Effect sizes for between-group comparisons were estimated using rank-biserial correlation. All *P*-values reported in correlation matrix figures are adjusted for multiple comparisons using Bonferroni correction.

In addition to the pre-specified analyses testing our main hypotheses—namely, the association between ARD and cognitive impairment, correlation comparisons, group-wise differences in ARD magnitude and a stepwise multiple regression of ARD on cognitive subdomains—we conducted several *post hoc* exploratory analyses. These included an EFA, subgroup-level correlations and correlations between ARD and patients’ depression, anhedonia, age, education and the length of the caregiver–patient relationship. We also performed an additional regression analysis predicting caregiver-rated apathy (AMI-CG) from self-reported apathy (AMI-SR) and cognitive performance. These analyses were intended to further characterize observed patterns.

## Results

### Prevalence of apathy, depression and anhedonia in neurological disorders

**Figure 1:**
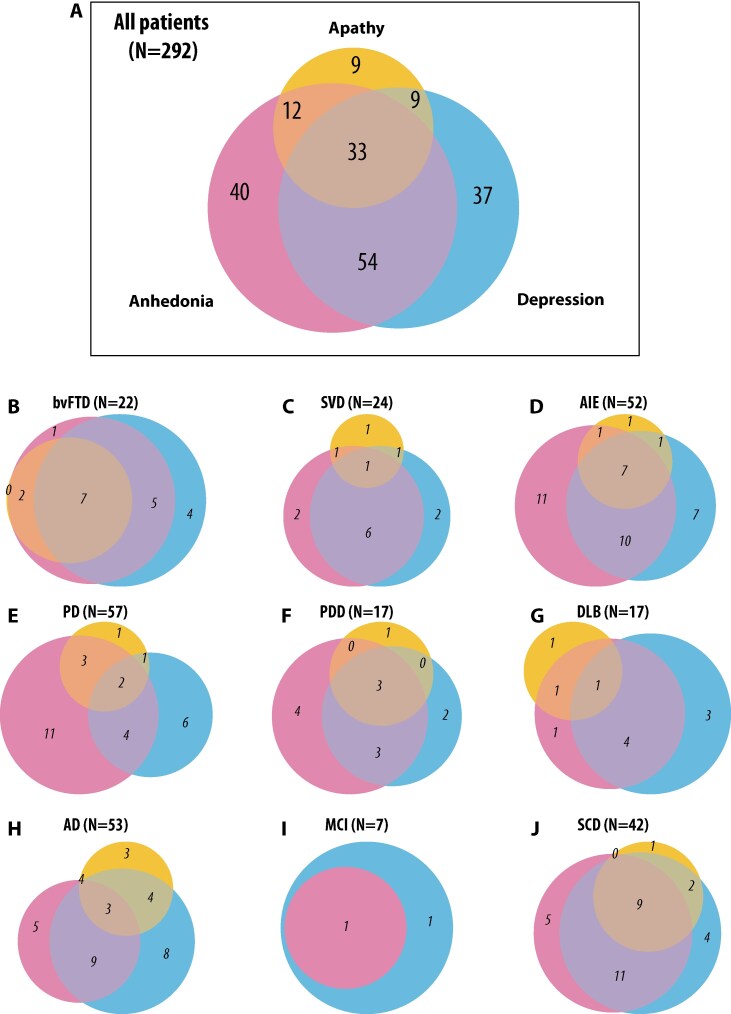
**Prevalence of self-reported apathy, depression, and anhedonia.** (**A**) Venn diagram illustrating the overlap between self-reported apathy, depression and anhedonia in all patients (*N* = 292). Yellow, pink and blue circles represent apathy, anhedonia and depression, respectively. See the ‘Methods’ section for cut-off criteria. The SD group is excluded due to insufficient data (*n* = 1). Details of prevalence are in Supplementary Table 2. (**B–J**) Venn diagrams illustrating the overlap between self-reported apathy, depression and anhedonia within each diagnostic group: (**B**) bvFTD (*N* = 22), (**C**) SVD (*N* = 24), (**D**) AIE (*N* = 52), (**E**) PD (*N* = 57), (**F**) PDD (*N* = 17), (**G**) DLB (*N* = 17), (**H**) AD (*N* = 53), (**I**) MCI (*N* = 7) and (**J**) SCD (*N* = 42).

We began by examining the prevalence of self-reported apathy and related neuropsychiatric syndromes—depression and anhedonia—in our patient cohorts. Patients were classified as apathetic based on their AMI-SR total score, depressed based on their GDS total score and anhedonic based on their SHAPS total score (see the ‘Methods’ section for cut-offs). The prevalence of each syndrome and their overlaps are illustrated in Fig. 1. Overall, across the 292 patients who completed all three questionnaires, 21.6% self-reported clinically significant apathy, 45.5% met criteria for depression self-reported significant depression symptoms and 47.6% exceeded the threshold for anhedonia. Considerable overlap was observed between these syndromes, with 11.3% of the 293 patients exceeding the threshold for all three (Fig. 1A). The prevalence of each syndrome within each diagnostic cohort is presented in Supplementary Table 2.

**Figure 2:**
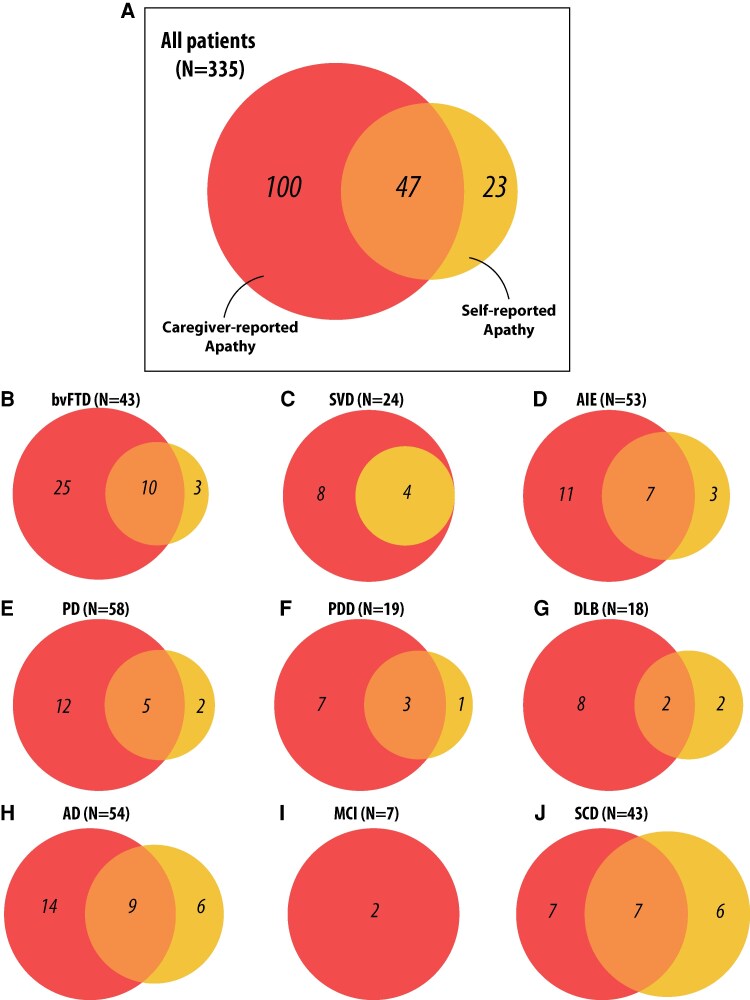
**Discrepancy in apathy determined by caregiver report and self-report.** Venn diagrams illustrating the discrepancy between caregiver- and self-reported apathy, using an AMI cut-off of 1.91 for both. Orange circles show apathy determined by caregiver report; yellow circles show apathy determined by self-report. (**A**) All patients (*N* = 335). (**B–J**) Discrepancy within each diagnostic group: (**B**) bvFTD (*N* = 43, (**C**) SVD (*N* = 24), (**D**) AIE (*N* = 53), (**E**) PD (*N* = 58), (**F**) PDD (*N* = 19), (**G**) DLB (*N* = 18), (**H**) AD (*N* = 54), (**I**) MCI (*N* = 7) and (**J**) SCD (*N* = 43). In the SD group (not shown), no individuals were classified as apathetic via self-report, whereas 6 of 16 individuals were classified as apathetic via caregiver report.

Focusing specifically on apathy, we compared the prevalence based on self-report and caregiver report in the 335 patients with both AMI-SR and AMI-CG data (Table 2). While 20.9% of patients self-reported apathy, the prevalence based on caregiver report was substantially higher, at 43.9% (Table 2 and Fig. 2). Although some agreement was observed between self- and caregiver report, discrepancies were evident in all neurological conditions except for SVD, where all patients identified as apathetic by self-report were also considered apathetic by their caregivers (Fig. 2). Across all conditions, the prevalence of apathy was consistently higher based on caregiver report than on self-report.

**Table 2:** Prevalence of self-reported versus caregiver-reported supra-threshold apathy across all diagnostic groups

Group	*N*	Self-reported apathy (%)	Caregiver-reported apathy (%)
bvFTD	43	30.2	81.4
SD	16	0.0	37.5
SVD	24	16.7	50.0
AIE	53	18.9	34.0
PD	58	12.1	29.3
PDD	19	21.1	52.6
DLB	18	22.2	55.6
AD	54	27.8	42.6
MCI	7	0.0	28.6
SCD	43	30.2	32.6
*All patients*	*335*	*20*.*9*	*43*.*9*
HC	19	0.0	5.3

This table includes all individuals who completed both the AMI-SR and AMI-CG, which only includes patients who also completed the GDS and SHAPS. Italics represents the total number of patients.

When investigating apathy, depression, and anhedonia prevalence using caregiver-reported AMI, we found a big increase in apathy prevalence in all groups (Supplementary Table 3). In summary, 292 patients across 10 neurological conditions had only 27.1% free from all three syndromes; in bvFTD, only 9.1% were free from these three syndromes. Apathy was present in 41.4% of all patients, depression in 45.5% and anhedonia in 47.6%, and 17.8% exhibited all three syndromes concurrently. Only 9.6% of patients presented with pure apathy, 10.6% with pure depression and 8.9% with pure anhedonia, highlighting that nearly one in five patients experience these conditions in isolation.

### Factor structure and reliability of the AMI-CG

**Figure 3:**
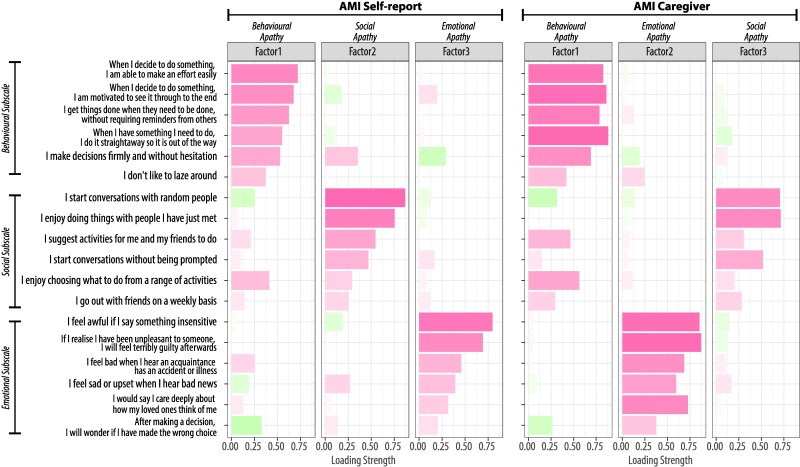
**Factor structures for AMI caregiver and AMI self-report (*N* = 335).** (Left) Results of the EFA for the 18-item AMI reported by patients themselves are shown for the three factors. The item label shows the original question used in the AMI-SR. The order of items is arbitrary. Pink bars indicate positive loadings, and green bars indicate negative loadings. Factors 1, 2 and 3 primarily load on questions from the three apathy subdomains of the AMI: behavioural apathy, social apathy and emotional apathy. The Kaiser–Meyer–Olkin measure was 0.85, confirming the sampling adequacy for factor analysis. Horn’s parallel analysis further supported the retention of three factors. EFA yielded a three-factor model that adequately fit the data [*χ*^2^(102) = 152.5, *P* < 0.001], explaining 37.5% of the variance. The model demonstrated good fit indices (RMSEA = 0.039, 90% CI 0.025–0.051; SRMR = 0.036; TLI = 0.94; CFI = 0.96; BIC = −439.3). This is consistent with the AMI-SR data from the prior studies in healthy population.^41^ (Right) The caregiver’s reports also assessed the same three apathy subdomains, but with a different factor order: behavioural apathy, emotional apathy and social apathy. The Kaiser–Meyer–Olkin measure was 0.90, confirming the sampling adequacy for factor analysis. Horn's parallel analysis further supported the retention of three factors. EFA yielded a three-factor model that adequately fit the data [*χ*^2^(102) = 229.4, *P* < 0.001], explaining 50.5% of the variance. The model demonstrated good fit indices (RMSEA = 0.062, 90% CI 0.051–0.072; SRMR = 0.035; TLI = 0.93; CFI = 0.95; BIC = −361.8). This structure, including the factor order, is consistent with the AMI-CG data from the prior study.^42^ Both exploratory factor analyses used parallel analysis to decide the number of factors. The factors appear non-orthogonal because Promax rotation was applied, allowing for inter-factor correlations. This choice was based on prior evidence that the subdomains of apathy are interrelated.

The three-factor structure of the AMI, previously identified in self-reported measures in healthy individuals^41^ and caregivers of Ad and PD patients,^42^ was replicated in our diverse clinical sample. EFA of AMI-SR (Fig. 3, left) and AMI-CG (Fig. 3, right) confirmed the presence of three distinct factors of apathy: behavioural inactivation, social disengagement, and emotional insensitivity.

The Kaiser–Meyer–Olkin measure was 0.90, confirming the sampling adequacy for factor analysis. Horn’s Parallel Analysis further supported the retention of three factors. EFA yielded a three-factor model that adequately fit the data [*χ*^2^(102) = 229.4, *P* < 0.001], explaining 50.5% of the variance. The model demonstrated good fit indices (RMSEA = 0.062, 90% CI 0.051–0.072; SRMR = 0.035; TLI = 0.93; CFI = 0.95; BIC = −361.8). Note that here EFA was conducted using Promax rotation, which allows for inter-factor correlations, based on prior evidence that the subdomains of apathy measure by the AMI are interrelated.^41,42^ While this approach ‘reduces’ factor orthogonality, the factor structure remained consistent when using Varimax rotation, confirming that the identified factors reflect meaningful constructs rather than an artefact of rotation choice. Therefore, we report the EFA results using Promax rotation.

The factor structure of the original AMI-SR was also confirmed in the AMI-CG, with Factor 1 (behavioural apathy), Factor 2 (emotional apathy) and Factor 3 (social apathy) loading onto corresponding items of the behavioural activation, emotional sensitivity and social motivation subscales, respectively (Fig. 3, right). The factors were intercorrelated (*r*_Behaviour-Emotional_ = 0.50, *r*_Behaviour-Social_ = 0.51 and *r*_Emotional-Social_ = 0.46).

The reliability and construct validity of the AMI-CG were also examined. Cronbach's alpha for the AMI-CG total score indicated good internal reliability (*α*_overall_ = 0.89, 95% CI 0.87–0.91). Internal consistency was good for the behavioural apathy (*α* = 0.87) and emotional apathy (*α* = 0.83) subscales and acceptable for the social apathy subscale (*α* = 0.74).

### Discrepancy between self and caregiver reports on apathy

Despite both patients and caregivers reporting on the same apathy items, only a moderate correlation was observed between the total AMI-SR and AMI-CG scores (*ρ* = 0.37, *P* < 0.001, *N* = 335). In contrast, this correlation was significantly stronger in HCs (*ρ* = 0.74, *P* < 0.001, *N* = 19; Fisher’s *z* = 2.20, *P* = 0.03), suggesting that among healthy individuals, self- and informant perceptions of apathy were generally aligned.

**Figure 4:**
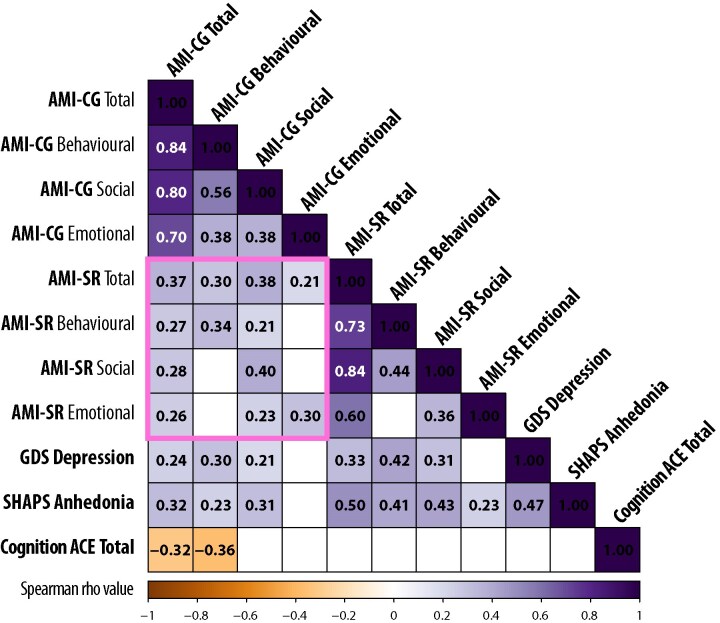
**Correlation matrix for AMI-CG and self-report measures.** Correlation matrix for AMI-CG total and subscale scores with self-reported AMI, GDS and SHAPS scores, along with ACE total score (cognitive function) for all patients (*N* = 335). The pink box highlights the crucial CG-SR discrepancy for AMI total score as well as the three subscales. Values in the cells and background colour indicate Spearman’s *ρ* correlation coefficients. Correlations that did not survive correction for multiple comparisons are left blank. Bonferroni correction was applied for multiple comparison correction with *α* = 0.00045, computed based on 0.05/total number of correlations computed.

Correlations within each subdomain were also significant but moderate, with the correlation found for social apathy (*ρ* = 0.40), behavioural apathy (*ρ* = 0.34) and emotional apathy (*ρ* = 0.30), all *P* < 0.001 (see Fig. 4). There were no significant differences in the strength of these correlations.

AMI-CG scores also significantly correlated with self-reported anhedonia (measured by SHAPS total score using 4-point scoring system, *ρ* = 0.33, *P* < 0.001; Fig. 4), which is considered a distinct construct. The total AMI-CG score in this study significantly correlated with self-reported depression (GDS; *ρ* = 0.25, *P* < 0.001). This remained significant even after removing the three apathy-related questions from the GDS (*ρ* = 0.24, *P* < 0.001).

These relationships between self-reported apathy, depression, and anhedonia were not unexpected, as these constructs often co-occur (Fig. 1).^3^ Such relationships have been seen in large-scale studies of healthy people throughout lifespan and are consistent with the notion that apathy and anhedonia may share overlapping features.

Given that self-reported apathy scores were generally lower than caregiver-reported scores, we sought to determine whether this discrepancy reflects a systematic reporting bias (i.e. patients consistently rate their apathy lower while still being sensitive to variations in their own apathy levels) or whether patients and caregivers differ in their perception of apathy in a more complex way. Specifically, we tested whether self-reported apathy across different domains (behavioural, social and emotional) could still predict caregiver-reported total apathy scores. To quantify this potential agreement between self-reported and caregiver-reported apathy, we conducted a multiple linear regression analysis. The self-reported scores on the three AMI subscales (behavioural, social and emotional) were entered as predictors of the caregiver-reported AMI total score. This model revealed that self-reported apathy, across all three domains, significantly predicted caregiver-reported total apathy [*F*(3, 331) = 12.97, *P* < 0.001, *R*^2^ = 0.141]. Each of the three subscales contributed significantly to the model (*t*_behavioural_ = 2.75, *P* = 0.006; *t*_social_ = 2.93, *P* = 0.004; *t*_emotional_ = 2.90, *P* = 0.004). However, despite this ‘significant’ prediction, the three self-report subscales accounted for only 14.1% of the variance in caregiver-reported total apathy scores.

This, combined with the moderate positive correlation found between AMI-SR and AMI-CG mentioned above, suggests that while there is partial agreement, there is also a clear divergence in how these two groups perceive and interpret patients’ apathetic behaviour. While a patient’s self-reported apathy provides some information about how their caregiver might perceive their apathy, it leaves most of the variance in caregiver report unexplained. This suggests that other factors, beyond the patient's own perception of their apathy, influence how caregivers perceive and rate apathy.

**Figure 5:**
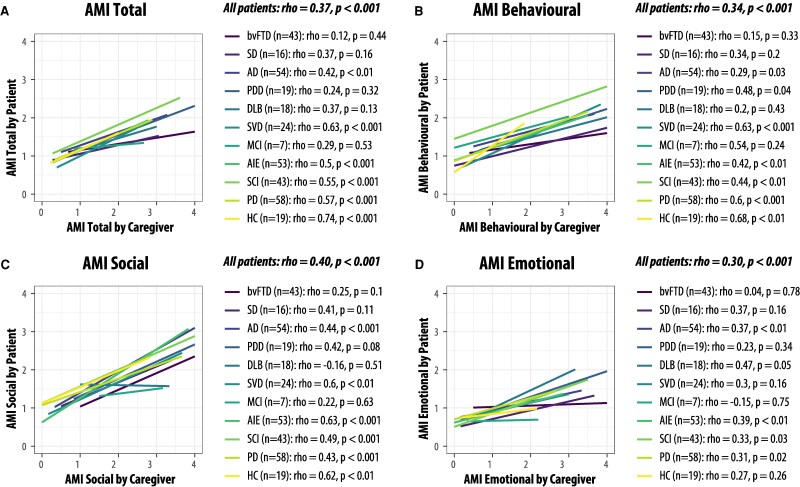
**Correlation between self-reported and caregiver-reported apathy across patient groups.** AMI-CG scores show moderate to strong correlations with their corresponding self-report scores in some, but not all, groups. (**A**) AMI total score, (**B**) AMI behavioural subscale, (**C**) AMI social subscale and (**D**) AMI emotional subscale. Coloured lines indicate each cohort. Spearman’s *ρ* and *P*-values are shown.

Further investigation of the correlations within each diagnostic group revealed a striking pattern: self- and caregiver-reported AMI total scores were strongly and positively correlated in SVD (*ρ* = 0.63), PD (*ρ* = 0.57), SCD (*ρ* = 0.55) and AIE (*ρ* = 0.50) (all *P* ≤ 0.001). In Ad, although self- and caregiver-reported scores showed a significant correlation, the relationship was moderate (*ρ* = 0.42, *P* = 0.002) (Fig. 5A–D). In contrast, no significant correlation was observed in bvFTD, SD, PDD, DLB or MCI. Notably, bvFTD exhibited the most pronounced lack of agreement, with no correlation between self- and caregiver-reported scores (*ρ* = 0.12, *P* = 0.43). This lack of alignment in bvFTD was consistent across all three domains of apathy: behavioural (*ρ* = 0.15, *P* = 0.34), social (*ρ* = 0.25, *P* = 0.10) and emotional (*ρ* = 0.07, *P* = 0.66).

### Underestimation of apathy by patients with neurological conditions

**Figure 6:**
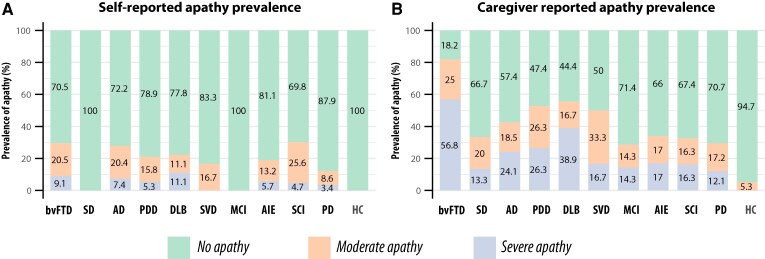
**Prevalence of apathy based on patient and caregiver reports.** The prevalence of apathy in different severity level is stacked as 100% for each group. (**A**) Apathy prevalence based on patient-reported AMI. (**B**) Apathy prevalence based on caregiver-reported AMI. The green portion of the bar represents no apathy (AMI total < 1.91), orange indicates moderate apathy (AMI total score ≥ 1.91) and purple indicates severe apathy (AMI total score ≥ 2.38). Sample sizes for each subgroup: bvFTD = 43, SD = 16, SVD = 24, AIE = 53, PD = 58, PDD = 19, DLB = 18, Ad = 54, MCI = 7, SCD = 43 and HCs = 19. See Supplementary Table 4 for the breakdown for each of three apathy subdomains.

To further understand this discrepancy, we examined the severity of apathy across different neurological conditions. Using established cut-offs on the AMI, we categorized apathy severity as none, moderate or severe based on both self-report and caregiver report (Fig. 6A and B). (We define ‘moderate apathy’ as apathy scores equal to or greater than one standard deviation above the mean AMI total score in a healthy population, i.e. AMI total score ≥1.91, and ‘severe apathy’ as scores exceeding two standard deviations, i.e. AMI total score ≥2.38. This terminology was previously used in Ang *et al*.^41^ and we adopt the same classification here.) Of 335 patients, 14.0% self-reported moderate apathy, with a statistically similar prevalence based on caregiver report (17.9%, *χ*2 = 1.6, *P* = 0.2). However, caregiver reports yielded a significantly higher prevalence of severe apathy across all conditions (self-reported severe apathy 5.4% versus caregiver-reported severe apathy 23.9%, *χ*^2^ = 44.5, *P* < 0.001). This underestimation by patients themselves was particularly pronounced in the bvFTD group, where self-reported severe apathy was only 9%, while caregiver reports indicated 58% with severe apathy (see more details in Supplementary Table 4).

**Figure 7:**
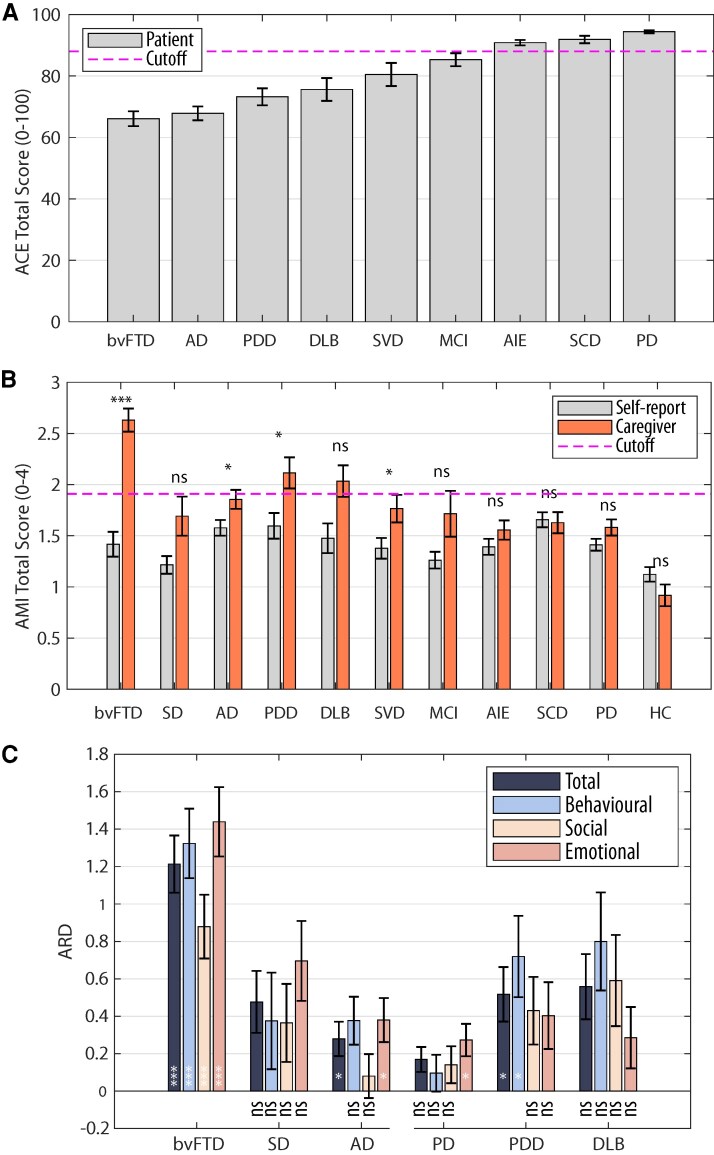
**Caregiver-reported apathy and cognitive impairment.** (**A**) Mean ACE total score for each cohort. The red horizontal dashed line indicates the cut-off for normal (88/100) ACE total score. Error bar indicates 1 SEM. (**B**) Mean AMI total score for each cohort rated by patients themselves (grey bars) and their caregivers (orange bars). Results of paired *t*-tests comparing self- and caregiver-reported AMI total scores are shown above the paired bars for each cohort. Bonferroni correction has been applied for multiple comparisons. (**C**) The ARD for AMI total, behavioural, social and emotional subscore. In all plots, error bars indicate 1 SEM. Results of one-sample *t*-test against 0 is shown at the bottom of each bar. Bonferroni correction has been applied for multiple comparisons. *P < 0.05, **P < 0.01, ***P < 0.001. ns, not significant. *P*-values were unadjusted. Sample sizes for each subgroup: bvFTD = 43, SD = 16, SVD = 24, AIE = 53, PD = 58, PDD = 19, DLB = 18, Ad = 54, MCI = 7, SCD = 43 and HCs = 19.

To explore the potential influence of cognitive impairment on the discrepancy, we ranked the patient groups by their average cognitive performance on the ACE-III (Fig. 7A). This analysis revealed a general trend for greater caregiver–self discrepancy in cohorts with poorer cognition (Fig. 7B; see Supplementary Fig. 1 for discrepancy in apathy subscales). However, the discrepancy in bvFTD was significantly greater than in any other neurological condition, including Ad, which had a similar level of cognitive impairment (no difference between their group ACE total score: *U* = 673.5, *P* = 0.46, ranksum *r* = 0.42). This finding suggests that factors beyond global cognitive impairment, such as lack of insight, may contribute to the underestimation of apathy in bvFTD.

Notably, the discrepancy between caregiver and self-report was significant larger in PDD and DLB than PD (ARD in PDD versus PD: *U* = 358.5, ranksum *r* = 0.35, *P* = 0.02; DLB versus PD: U = 355, ranksum *r* = 0.32, *P* = 0.04; Fig. 7C). In fact, the discrepancy in PD was mainly driven by a difference in emotional apathy, as there were no significant differences in apathy reporting in behavioural or social apathy (Fig. 7C). Likewise, a significant discrepancy was observed in Ad, but not in MCI or SCD (Fig. 7B).

### Caregiver–self apathy discrepancy and cognition

**Figure 8:**
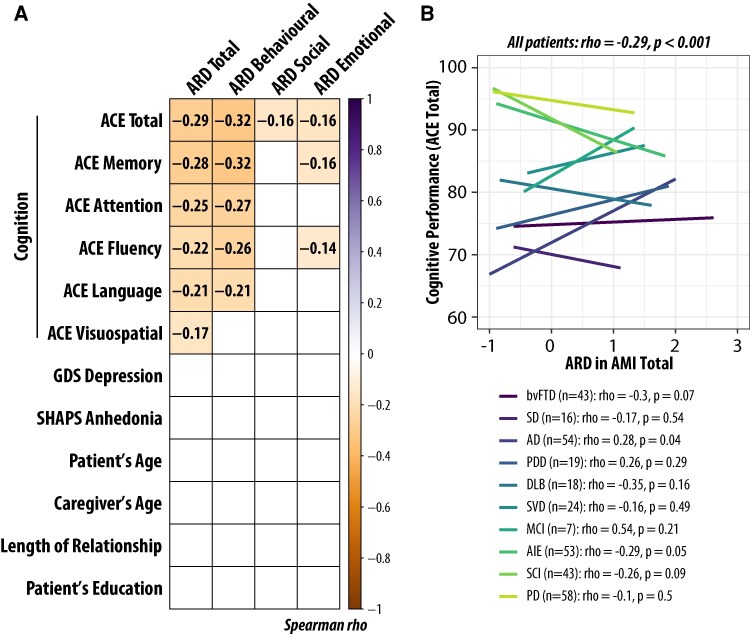
**Correlation between ARD and cognitive measures.** (**A**) Correlation matrix displaying the relationships between ARD, cognitive measures, depression, anhedonia and demographic variables across all patients (*N* = 314). Values in the cells and background colour indicate Spearman’s *ρ* correlation coefficients. Correlations that did not survive correction for multiple comparisons are left blank. Bonferroni correction was applied for multiple comparison correction with *α* = 0.001, computed based on 0.05/total number of correlations computed. (**B**) Correlation between ARD total and ACE total (i.e. the top left corner in **A**) across patient groups. Coloured lines indicate each cohort. Spearman’s *ρ* and *P*-values are shown.

To investigate the factors underlying the discrepancy between caregiver- and self-reported apathy, we calculated the ARD for each participant. ARD was defined as the difference between the AMI total score reported by the caregiver and the AMI total score reported by the patient (ARD = AMI_CG_ − AMI_SR_). Based on the subscale reported, we can also have ARD_Behavioural_ (i.e. discrepancy in AMI behavioural subscale), ARD_Social_ and ARD_Emotional_. Thus, ARD quantifies the extent to which caregivers perceive patients as more apathetic than the patients perceive themselves. Figure 8A presents a correlation matrix examining the relationships between ARD (total score and subscale scores) and various factors, including cognitive measures (ACE-III total and subdomain scores), depression (self-reported GDS depression-related items’ mean score, see the ‘Methods’ section for rationale behind this choice), anhedonia (self-reported SHAPS total score), patient age, caregiver age and education level.

Our analysis revealed that ARD could not be explained by patient depression (GDS depression score, *ρ* = 0.007, *P* = 0.9, *N* = 295), anhedonia level (SHAPS total score, *ρ* = −0.050, *P* = 0.4, *N* = 299), patient or caregiver age (*ρ* < 0.08, *P* > 0.2), length of the caregiver–patient relationship (*ρ* = 0.05, *P* = 0.4, *N* = 293) or patient education level (*ρ* = −0.12, *P* = 0.05, *N* = 259; this correlation did not survive correction for multiple comparisons).

Among the 314 patients with both ACE and AMI data (including ACE-R data from the SD group), ARD significantly negatively correlated with overall cognitive function (ACE total score; *ρ* = −0.29, *P* < 0.001). There are several possible explanations for this negative correlation between ARD and cognitive impairment: for example cognitively impaired patients may underestimate their own apathy or fail to understand and respond to questionnaires meaningfully, or caregivers of patients with cognitive deficits endorse apathy items more commonly (possibly due to caregiver burden). The correlations between ARD and ACE scores within individual diagnostic groups should be interpreted with caution due to limited statistical power. Detecting a moderate correlation (*ρ* ≥ 0.3) with *α* = 0.05 and *β* = 0.20 requires an estimated sample size of 85, which none of the subgroups achieved. Subgroup-level correlations are nonetheless provided in the figure legend for reference (Fig. 8B).

The negative relationship between ARD and cognition was seen for all types of apathy, although with variation in the strength of correlation: behavioural apathy (*ρ* = −0.32, *P* < 0.001), with weak correlations with social apathy (*ρ* = −0.16, *P* = 0.005) and emotional apathy (*ρ* = −0.16, *P* = 0.004). ARD in behavioural apathy was associated with variation in all cognitive subdomains of the ACE: memory performance (*ρ* = −0.32, *P* < 0.001), and weakly associated with attention (*ρ* = −0.27, *P* < 0.001), verbal fluency (*ρ* = −0.26, *P* < 0.001), language (*ρ* = −0.21, *P* < 0.001) and visuospatial perception (ACE visuospatial) (*ρ* = −0.16, *P* = 0.005). The correlation between cognitive function and ARD_Behavioural_ was strongest, significantly higher than both ARD_Social_ (z = −2.84, *P* = 0.002) and ARD_Emotional_ (z = −2.44, *P* = 0.007).

To further examine the relationship between cognitive function and the discrepancy between caregiver and self-reported apathy (ARD), a stepwise multiple linear regression analysis was conducted using all five ACE subscores as predictors. Two models significantly predicted ARD across all patients: (i) Model 1 included only the memory subscore and was statistically significant, *F*(1312) = 31.4, *P* < 0.001, explaining 9.2% of the variance in ARD (*R*^2^ = 0.092). (ii) Model 2 included both the memory and fluency subscores and was also statistically significant, *F*(2311) = 18.9, *P* < 0.001, explaining 10.8% of the variance in ARD (*R*^2^ = 0.108). These results suggest that memory function alone is a significant predictor of caregiver–self apathy discrepancy, but adding fluency as a predictor provides a modest increase in explained variance (see Supplementary Materials for detailed modelling results).

Furthermore, we re-ran the multiple linear regression on the subsample of 313 patients predicting total AMI-CG from AMI-SR subscales and cognition (ACE) data. We started with the model without cognition, which shows as previously that, the three self-reported AMI subscales explained 14.3% of the variance in caregiver-rated AMI total score [*P* < 0.001, *F*(3, 310) = 17.3, BIC = 687], with all three subscales contributing significantly (*P* ≤ 0.019, *t* ≥ 2.36).

To assess the contribution of specific cognitive domains, we compared this null model (three self-reported subscales) to a full model incorporating all ACE subscores (memory, attention, fluency, language, and visuospatial). Two models significantly improved upon the null model. The first model included, in addition to the three self-reported AMI subscales, the ACE fluency score. The model explained an additional 9.3% of the variance [total variance explained = 23.6%; *F* change (1309) = 37.5, *P* < 0.001, BIC = 657.1]. The substantial decrease in BIC (ΔBIC = 30.3) strongly suggests that the model incorporating the fluency performance provides a better fit to the data. The ACE fluency score contributed significantly (*P* < 0.001, *t* = −6.13), with a larger standardized coefficient value (−0.31) than all three self-reported AMI subscales (standardized coefficients ≤ 0.17). The second model included the ACE-III memory subscore in addition to fluency. Compared with the first model, the addition of ACE-III memory explained an additional 1.9% of the variance [total variance explained = 25.5%; *F* change (1, 308) = 7.95, *P* = 0.005, BIC = 654.9]. Overall, these findings indicate that patients with greater cognitive impairment, particularly in verbal fluency and memory, exhibit a larger discrepancy between caregiver- and self-reported apathy (see Supplementary Materials for full modelling results).

## Discussion

This study revealed a significant discrepancy between self- and caregiver-reported apathy across multiple neurological disorders: bvFTD, SD, SVD, PD, PDD, DLB, Ad, MCI, AIE and SCD (Figs 5 and 7B). This discrepancy was observed across all apathy subdomains—behavioural, social and emotional (Fig. 7C)—and was more pronounced in conditions with greater cognitive impairment. For example, PDD and DLB demonstrated larger discrepancies than PD with normal cognitive performance. Similarly, the discrepancy was greater in bvFTD than Ad, likely reflecting the more severe lack of insight associated with bvFTD despite similar scores on a global cognitive screening task (ACE). These findings underscore the critical importance of considering both patient and caregiver reports for accurately assessing apathy in neurological conditions.

An extensive literature has demonstrated that apathy is common in all types of dementia, with prevalence ranging from one-third to almost 100%.^63^ For example, although apathy is commonly reported to be more severe in bvFTD compared to SD and Ad,^15,64,65^ the reported prevalence in bvFTD still ranges widely from 50% to 90% across different studies.^15,43-46,64-71^ This variability may rise from the use of different screening tools, operational definitions of apathy or thresholding approaches.^63^ Investigations using apathy-specific scales like Starkstein’s Apathy Scale or Apathy Evaluation Scale generally report higher prevalence rates compared to those conducted with the neuropsychiatric inventory (NPI).^63,72^

In our study, self-reported data initially suggested a lower prevalence of apathy than expected. For instance, only 30% of bvFTD patients self-reported apathy on the AMI (Table 2), significantly below the 50–90% range reported in the literature using other scales.^63^ Further investigation revealed that this may be attributable to patient underreporting or the cut-off thresholds used. When caregiver-reported AMI scores were considered, 81% of bvFTD patients were apathetic, with 58% classified as severely apathetic (Table 2). Prevalence rates for other conditions in our study align closely with prior literature: 37% for SD, 50% for SVD,^73^ 44% for AIE^74^ and 29% for PD.^75^ As expected, apathy prevalence was higher in PDD (52%) and DLB (56%) than in PD.^76-78^ Consistent with prior findings, Ad showed greater apathy prevalence (43%) compared to MCI (29%) and SCD (33%).^79,80^ In summary, these findings reveal the limitations of relying solely on self-reports for diagnosing apathy, particularly in older adults and those with severe apathy.

Another important consideration is the use of cut-off thresholds in assessment tools for identifying apathy. Apathy exists along a continuous spectrum, varying in severity across one or more dimensions, rather than being a binary condition that is simply present or absent. However, it is common practice to define apathy based on a predetermined severity threshold. Thresholds derived from younger, healthy populations^41^ may not be suitable for older adults. Our unpublished AMI data from 1361 healthy adults aged over 50 years suggest an adjusted cut-off (AMI total ≥1.72). Using this adjustment, caregiver-reported prevalence increased to 91% in bvFTD and 79% in PDD. At the same time, such adjustment would not affect the apathy prevalence in the healthy control group. These findings underscore the need for standardization in apathy assessment tools for more accurate and consistent evaluations in dementia research.

Previous reports have consistently shown that caregivers report higher levels of apathy in patients than patients report themselves.^15,21,24,27,30-32,42,81-83^ Without an objective measure of goal-directed behaviour, it remains unclear whether patients underreport their symptoms or caregivers overreport them. Individuals with apathy may perceive themselves as ‘content’ and be less troubled by behavioural changes, whereas caregiver burden may heighten sensitivity to signs of apathy. Regardless of the underlying case, this discrepancy is striking—self-reported AMI scores for behavioural, social and emotional domains together explained ‘only’ 14.1% of the variance in caregiver-reported AMI scores. More importantly, we found that this discrepancy also varied significantly across disorders (Figs 5 and 7). In conditions such as SVD, PD, SCD and AIE, self and caregiver ratings were strongly correlated, suggesting that the discrepancy reflected differences in severity rather than a fundamental disconnect. However, in conditions with greater cognitive impairment, such as PDD, DLB, Ad and FTD, the correlation was weaker or even non-significant.

Here, patients’ cognitive impairment emerged as a key factor influencing the magnitude of the ARD. Specifically, across conditions, greater cognitive impairment was associated with larger discrepancies. The positive relationship between ARD and patients’ cognitive impairment reported here cannot be solely attributed to a general caregiver reporting bias linked to increased caregiver burden. If caregiver burden were the primary driver, we would expect an overall inflation in caregiver-reported apathy scores equally across all apathy domains. However, as shown in Fig. 8A, the relationship between ARD and cognition was specific to the behavioural AMI subscale, with the discrepancy being particularly pronounced in patients with greater impairments in memory, attention and fluency. This suggests that cognitive deficits may selectively impact how patients perceive and report their own apathy, rather than caregivers simply overestimating apathy across the board.

Moreover, patients’ memory impairment and fluency deficits explained an additional 11.2% of the variance in caregiver-reported apathy. These findings are consistent with the idea that impairments in executive and memory domains contribute to reduced awareness of behavioural change. Impaired fluency may restrict patients’ ability to generate or consider different actions spontaneously, resulting in inertia.^84^ Additionally, memory deficits could prevent patients from following through with plans or recalling past intentions, further reinforcing the caregiver’s perception of reduced motivation. Together, these may explain the greater discrepancy between caregiver- and self-reported apathy in cognitively impaired individuals. Another possible explanation is that patients with greater memory impairment may simply forget their own levels of behavioural inactivity, leading them to underestimate their apathy. In contrast, caregivers may provide a more accurate account of apathetic episodes. Thus, rather than simply reflecting caregiver overestimation as proposed in previous studies,^27,30,31,85^ the observed discrepancy between self- and caregiver-reported apathy may stem from distinct cognitive mechanisms, specifically deficits in memory and executive function, that shape how patients perceive and experience apathy across different neurological conditions.

Among conditions with similar cognitive screening total score, such as bvFTD and Ad, the discrepancy was larger in bvFTD. (Note that the pattern of cognitive deficits over domains differs markedly in the ACE, and only the total scores are comparable.) This suggests the role of impaired insight in these patients on the ARD. In this group, the lack of agreement between self- and caregiver reports was consistent across all three apathy domains, and the magnitude of the discrepancy was particularly striking. Cognitive impairment alone cannot fully explain this discrepancy, as Ad patients, despite comparable cognitive deficits, demonstrated smaller discrepancies than bvFTD patients, consistent with previous reports on bvFTD and Ad.^21,64,86^ This pattern aligns with research on anosognosia in dementia, where discrepancies between self- and caregiver reports of cognitive deficits have been linked to patients’ diminished self-awareness.^29,38,85,87^ In such cases, greater divergence between caregiver and patient ratings reflects more severe reductions in patients’ awareness.^85,88^

We also investigated whether patients’ depression or anhedonia could explain the ARD, because apathy, depression and anhedonia are very relevant in both healthy and patient population.^3,89^ Despite the frequent co-occurrence of apathy, depression and anhedonia, our findings suggest that the underestimation of apathy by patients cannot simply be attributed to low mood or an inability to experience pleasure of patients themselves. This aligns with a previous study that found caregivers’ depression did not account for discrepancies in apathy reporting either.^30^ These findings reinforce that the discrepancy in apathy reporting is unlikely to result from negative emotional bias in patients or caregivers but rather reflects more complex factors, such as impaired patient awareness and cognitive impairment. In addition to these factors, caregiver burden could be another critical issue, though this was not investigated in the present study. Previous studies have shown that caregiver burden correlated with patients’ cognitive impairment and predicted the ARD.^21,30,31,47^ For instance, caregivers’ ratings of apathy increased over time in Ad and MCI patients, even when patients’ self-reports remained stable.^31^

### Limitations

While our findings are robust, several limitations should be acknowledged. First, cohort sizes varied across diagnostic groups, potentially limiting statistical power. In particular, the MCI group was small and the specific results for this group should be interpreted with caution. Second, details on clinician-rated apathy or objective quantitative measure of apathy were not systematically collected in this study, limiting direct comparison with an independent reference. However, the AMI was adapted from the clinician-administered Lille Apathy Rating Scale interview,^41,90^ and AMI-CG demonstrated strong correlations with Lille Apathy Rating Scale (*ρ* = 0.72, *P* < 0.01), supporting the use of AMI as a proxy for clinician-rated apathy.^42^ The AMI has also been validated in both healthy populations (*N* > 4500)^89^ and patients with neurological conditions,^42,91^ further confirming its applicability across diverse cohorts. Third, we did not include a quantitative measure of impaired insight, such as Abridged Anosognosia Questionnaire–Dementia^92^ or Beck Cognitive Insight Scale^93^ (see a recent systematic evaluation of anosognosia measures^94^). Fourth, the study did not directly measure caregivers’ depression or perceived burden, which could have influenced their ratings. Fifth, we used categorized (thresholded) classification of apathy based on supra/infra-threshold endorsements of apathy items, rather than continuous severity variables. While this facilitates the comparison of groups, we are aware that apathy within the ‘normal’ range may still be relevant in the context of disease and its progression. Future research incorporating caregiver burden assessments might provide further understanding of contributions to apathy reporting discrepancies. Additionally, the proportion of caregivers in this study who were primary caregivers remains unclear.

## Conclusion

This study highlights the need to consider both patient and caregiver perspectives on apathy, especially for conditions with prominent cognitive impairment. To improve the recognition and management of apathy, and improve the understanding of the neurocognitive mechanisms of apathy, standardized assessment tools are required that are applicable to people with cognitive deficits. These will facilitate clinical interventions to benefit both patients and caregivers.

## Supplementary Material

fcaf235_Supplementary_Data

## Data Availability

The data supporting the findings of this study are available from the corresponding author upon reasonable request. No specialized in-house software or pipelines were developed for this work. All modelling was performed using JASP, which operates via a graphical interface and does not require scripting. Codes used for data visualization in R and MATLAB are available at https://osf.io/m78je/.
