# Supplementary material for: Self- versus caregiver-reported apathy across neurological disorders
Source: Brain Commun. 2025 Jun 13;7(3):fcaf235. doi: 10.1093/braincomms/fcaf235 (PMC12188441; doi:10.1093/braincomms/fcaf235)
Supplement: fcaf235_Supplementary_Data [file fcaf235_supplementary_data.docx]

## Table of Contents

[Supplementary Figure 1: Caregiver-reported apathy and cognitive impairment. 2](#_Toc195274263)

[Supplementary Table 1. Demographics and clinical characteristics of participants. 3](#_Toc195274264)

[Supplementary Table 2: Prevalence of self-reported apathy, depression and anhedonia. 4](#_Toc195274265)

[Supplementary Table 3: Prevalence of caregiver-reported apathy, self-reported depression and self-reported anhedonia. 5](#_Toc195274266)

[Supplementary Table 4: Prevalence of different apathy severity based on patient and caregiver reports. 6](#_Toc195274267)

[Modelling results 7](#_Toc195274268)

[Stepwise multiple linear regression predicting ARD from five ACE subscores 7](#_Toc195274269)

[Multiple linear regression predicting total AMI-CG from AMI-SR subscales and ACE 8](#_Toc195274270)

### ****Supplementary Figure 1: Cohort-wise comparison between AMI self-report and AMI caregiver.****

(A) Mean AMI Behavioural Apathy score for each cohort. The grey bar shows the mean of self-reported score and the orange shows the caregiver’s report. Results of paired t-tests comparing self- and caregiver-reported AMI total scores are shown above the paired bars for each cohort. **The same bar plot for** Social Apathy **(B)**, and Emotional Apathy **(C) AMI subscore**. In all plots, error bars indicate 1 SEM. Results of one-sample t-test against 0 is shown at the bottom of each bar. * p < 0.05, ** p < 0.01, *** p < 0.001. N.S. = not significant. p values were unadjusted. Sample sizes for each subgroup: bvFTD = 43, SD = 16, SVD = 24, AIE = 53, PD = 58, PDD = 19, DLB = 18, AD = 54, MCI = 7, SCD = 43, HC = 19.

**
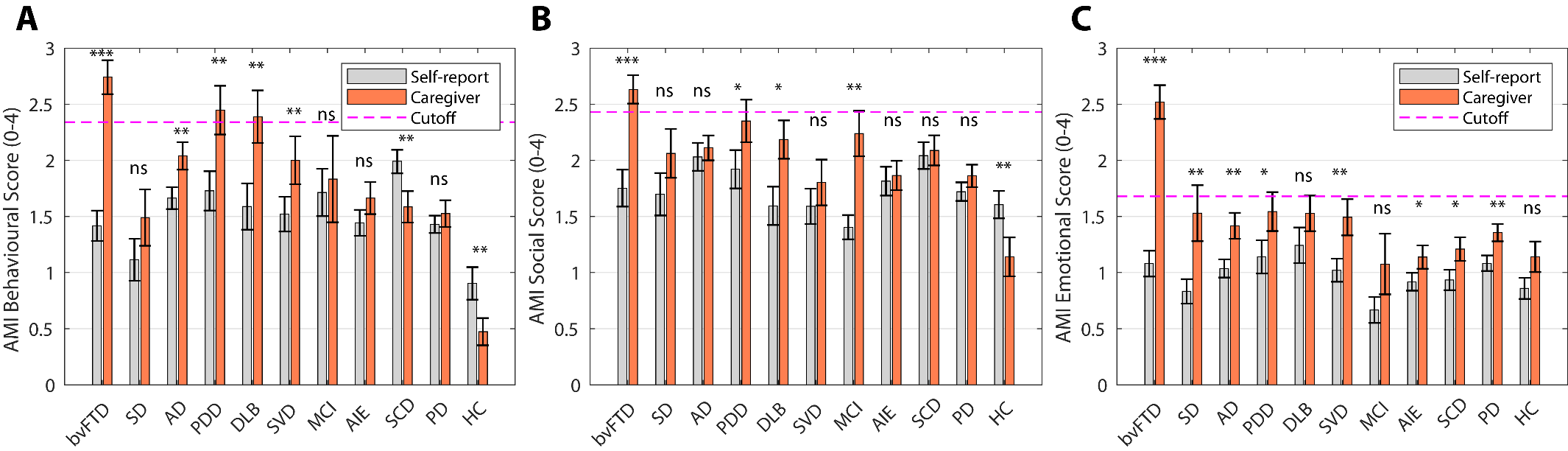
**

### Supplementary Table 1. Demographics and clinical characteristics of participants.

| **Diagnosis** | **N** | **Gender** | **Caregiver**  **Gender** | **Age** | **Caregiver Age** | **Length of**  **Relationship (years)** | **Education** | **ACE Total** | **AMI-SR**  **Total** | **AMI-CG**  **Total** |
| --- | --- | --- | --- | --- | --- | --- | --- | --- | --- | --- |
| bvFTD | 43 | F13 M30 | F15 M7 (N=22) | 62.95  (9.55, N=40) | 62.37  (10.93, N=19) | 44.55  (13.38, N=22) | 12.50 (2.41, N=14) | 66.11  (15.82, N=38) | 1.42  (0.79, N=43) | 2.63  (0.74, N=43) |
| SD | 16 | F11 M5 | * | 64.69  (7.43, N=16) | * | * | * | 59.81  (14.46, N=16) | 1.22 (0.35, N=16) | 1.69  (0.76, N=16) |
| SVD | 24 | F4 M20 | F17 M3 (N=20) | 70.08 (7.56, N=24) | 66.11 (7.29, N=18) | 42.91  (14.55, N=22) | 12.29  (2.81, N=21) | 80.50  (18.43, N=22) | 1.38  (0.50, N=24) | 1.77  (0.66, N=24) |
| AIE | 53 | F18 M35 | F20 M4 (N=24) | 63.38  (13.45, N=53) | 54.29  (16.40, N=14) | 36.10  (16.19, N=51) | 12.80  (3.34, N=50) | 90.85  (6.45, N=47) | 1.39  (0.57, N=53) | 1.56  (0.68, N=53) |
| PD | 58 | F23 M35 | F6 M1 (N=7) | 68.47  (7.63, N=58) | 59.43  (17.00, N=7) | 42.04  (15.20, N=57) | 15.75  (4.10, N=53) | 94.40  (3.53, N=53) | 1.41  (0.44, N=58) | 1.58  (0.60, N=58) |
| PDD | 19 | F4 M15 | F10 M1 (N=11) | 71.05  (7.55, N=19) | 66.67  (12.85, N=9) | 38.89  (14.52, N=18) | 12.50  (3.06, N=12) | 73.21  (11.97, N=19) | 1.60  (0.55, N=19) | 2.11  (0.66, N=19) |
| DLB | 18 | F4 M13 | F15 M3 (N=18) | 69.24  (7.23, N=17) | 61.41  (12.57, N=17) | 38.83  (17.54, N=18) | 14.40  (4.75, N=15) | 75.61  (15.81, N=18) | 1.48  (0.62, N=18) | 2.03  (0.65, N=18) |
| AD | 54 | F24 M30 | F16 M5 (N=21) | 69.33  (9.39, N=54) | 60.33  (14.87, N=12) | 42.49  (14.29, N=53) | 14.21  (3.64, N=47) | 67.82  (16.61, N=51) | 1.58  (0.56, N=54) | 1.86  (0.68, N=54) |
| MCI | 7 | F6 M1 | F2 M1 (N=3) | 64.57 (6.90, N=7) | * | 42.14 (10.33, N=7) | 12.57 (2.88, N=7) | 85.29 (5.59, N=7) | 1.26 (0.22, N=7) | 1.72 (0.59, N=7) |
| SCD | 43 | F20 M23 | F8 M3 (N=11) | 57.02  (9.98, N=43) | 61.40  (8.11, N=5) | 30.74  (14.67, N=43) | 14.95  (3.66, N=38) | 91.88  (7.89, N=43) | 1.66  (0.48, N=43) | 1.63  (0.68, N=43) |
| HC | 19 | F11 M8 | * | 64.26  (6.71, N=19) | * | * | * | 97.16 (1.57, N=19) | 1.12  (0.31, N=19) | 0.92  (0.46, N=19) |
| *All patients* | 335 | F127 M207 | F109 M28 (N=137) | 65.68  (10.39, N=331) | 61.66  (12.68, N=102) | 39.24 (15.41, N=292) | 13.99  (3.77, N=258) | 80.48  (17.04, N=314) | 1.47  (0.56, N=335) | 1.84  (0.75, N=335) |

For continuous variables (age, length of patient-caregiver relationship, etc.), the mean and standard deviation (SD) are reported, along with the number of participants for whom this information was available (indicated in parentheses).

Abbreviations: ACE-III = Addenbrooke's Cognitive Examination-III. AMI-SR = Apathy-Motivation Index Self-reported version. AMI-CG = Apathy-Motivation Index caregiver version. bvFTD = behavioural variant frontotemporal dementia; SD = semantic dementia; SVD = small vessel disease; AIE = autoimmune encephalitis; PD = Parkinson's disease; PDD = Parkinson's disease dementia; DLB = dementia with Lewy bodies; AD = Alzheimer's disease; MCI = mild cognitive impairment; SCD = subjective cognitive decline; HC = healthy controls.

^a^"All patients" refers to all diagnostic groups combined, excluding healthy controls.

* = data missing.

### Supplementary Table 2: Prevalence of self-reported apathy, depression and anhedonia.

| **Group** | **N** | **SR Apathy** | **Depression** | **Anhedonia** | **No ADA** | **ADA** | **Apathy &**  **Depression** | **Apathy &**  **Anhedonia** | **Depression &**  **Anhedonia** | **Pure**  **Apathy** | **Pure**  **Depression** | **Pure**  **Anhedonia** |
| --- | --- | --- | --- | --- | --- | --- | --- | --- | --- | --- | --- | --- |
| bvFTD | 22 | 40.9% | 72.7% | 68.2% | 13.6% | 31.8% | 0.0% | 9.1% | 22.7% | 0.0% | 18.2% | 4.5% |
| SVD | 24 | 16.7% | 41.7% | 41.7% | 41.7% | 4.2% | 4.2% | 4.2% | 25.0% | 4.2% | 8.3% | 8.3% |
| AIE | 52 | 19.2% | 48.1% | 55.8% | 26.9% | 13.5% | 1.9% | 1.9% | 19.2% | 1.9% | 13.5% | 21.2% |
| PD | 57 | 12.3% | 22.8% | 35.1% | 50.9% | 3.5% | 1.8% | 5.3% | 7.0% | 1.8% | 10.5% | 19.3% |
| PDD | 17 | 23.5% | 47.1% | 58.8% | 23.5% | 17.6% | 0.0% | 0.0% | 17.6% | 5.9% | 11.8% | 23.5% |
| DLB | 17 | 17.6% | 47.1% | 41.2% | 35.3% | 5.9% | 0.0% | 5.9% | 23.5% | 5.9% | 17.6% | 5.9% |
| AD | 53 | 26.4% | 45.3% | 39.6% | 32.1% | 5.7% | 7.5% | 7.5% | 17.0% | 5.7% | 15.1% | 9.4% |
| MCI | 7 | 0.0% | 28.6% | 14.3% | 71.4% | 0.0% | 0.0% | 0.0% | 14.3% | 0.0% | 14.3% | 0.0% |
| SCD | 42 | 28.6% | 61.9% | 59.5% | 23.8% | 21.4% | 4.8% | 0.0% | 26.2% | 2.4% | 9.5% | 11.9% |
| All patients | 292 | 21.6% | 45.5% | 47.6% | 33.6% | 11.3% | 3.1% | 4.1% | 18.5% | 3.1% | 12.7% | 13.7% |

The percentage was computed based on all patients (N=292) in that diagnostic group who completed all three questionnaires: AMI for apathy, GDS for depression, and SHAPS for anhedonia. 'No ADA' refers to the proportion of individuals who did not experience apathy, depression, or anhedonia. 'ADA' indicates those who experienced all three conditions—apathy, depression, and anhedonia.Only one patient in the Semantic Dementia (SD) group completed all three questionnaires (AMI, GDS, and SHAPS), so the prevalence for this group is not reported here. bvFTD = behavioural variant frontotemporal dementia; SD = semantic dementia; SVD = small vessel disease; AIE = autoimmune encephalitis; PD = Parkinson's disease; PDD = Parkinson's disease dementia; DLB = dementia with Lewy bodies; AD = Alzheimer's disease; MCI = mild cognitive impairment; SCD = subjective cognitive decline; HC = healthy controls. Visualisation is in Figure 1.

### Supplementary Table 3: Prevalence of caregiver-reported apathy, self-reported depression and self-reported anhedonia.

| **Group** | **N** | **CG Apathy** | **Depression** | **Anhedonia** | **No ADA** | **ADA** | **Apathy &**  **Depression** | **Apathy &**  **Anhedonia** | **Depression &**  **Anhedonia** | **Pure**  **Apathy** | **Pure**  **Depression** | **Pure**  **Anhedonia** |
| --- | --- | --- | --- | --- | --- | --- | --- | --- | --- | --- | --- | --- |
| bvFTD | 22 | 72.7% | 72.7% | 68.2% | 9.1% | 45.5% | 13.6% | 9.1% | 9.1% | 4.5% | 4.5% | 4.5% |
| SVD | 24 | 50.0% | 41.7% | 41.7% | 29.2% | 20.8% | 4.2% | 8.3% | 8.3% | 16.7% | 8.3% | 4.2% |
| AIE | 52 | 34.6% | 48.1% | 55.8% | 23.1% | 15.4% | 3.8% | 9.6% | 17.3% | 5.8% | 11.5% | 13.5% |
| PD | 57 | 29.8% | 22.8% | 35.1% | 47.4% | 10.5% | 1.8% | 12.3% | 0.0% | 5.3% | 10.5% | 12.3% |
| PDD | 17 | 52.9% | 47.1% | 58.8% | 17.6% | 29.4% | 11.8% | 0.0% | 5.9% | 11.8% | 0.0% | 23.5% |
| DLB | 17 | 52.9% | 47.1% | 41.2% | 23.5% | 23.5% | 5.9% | 5.9% | 5.9% | 17.6% | 11.8% | 5.9% |
| AD | 53 | 43.4% | 45.3% | 39.6% | 20.8% | 9.4% | 5.7% | 11.3% | 13.2% | 17.0% | 17.0% | 5.7% |
| MCI | 7 | 28.6% | 28.6% | 14.3% | 42.9% | 0.0% | 0.0% | 0.0% | 14.3% | 28.6% | 14.3% | 0.0% |
| SCD | 42 | 33.3% | 61.9% | 59.5% | 23.8% | 19.0% | 4.8% | 7.1% | 28.6% | 2.4% | 9.5% | 4.8% |
| All patients | 292 | 41.4% | 45.5% | 47.6% | 27.1% | 17.8% | 5.1% | 8.9% | 12.0% | 9.6% | 10.6% | 8.9% |

The percentage was computed based on all patients (N=292) in that diagnostic group who completed all three questionnaires: AMI for apathy, GDS for depression, and SHAPS for anhedonia. 'No ADA' refers to the proportion of individuals who did not experience apathy, depression, or anhedonia. 'ADA' indicates those who experienced all three conditions—apathy, depression, and anhedonia.Only one patient in the Semantic Dementia (SD) group completed all three questionnaires (AMI, GDS, and SHAPS), so the prevalence for this group is not reported here.

bvFTD = behavioural variant frontotemporal dementia; SD = semantic dementia; SVD = small vessel disease; AIE = autoimmune encephalitis; PD = Parkinson's disease; PDD = Parkinson's disease dementia; DLB = dementia with Lewy bodies; AD = Alzheimer's disease; MCI = mild cognitive impairment; SCD = subjective cognitive decline; HC = healthy controls.

### Supplementary Table 4: Prevalence of different apathy severity based on patient and caregiver reports.

| **Group** | **Behavioural Apathy** | | **Social Apathy** | | **Emotional Apathy** | |
| --- | --- | --- | --- | --- | --- | --- |
|  | SR | CG | SR | CG | SR | CG |
| bvFTD | 86/9/5 | 40/14/47 | 79/5/16 | 44/30/26 | 81/9/9 | 23/16/60 |
| SD | 94/6/0 | 88/6/6 | 75/25/0 | 75/12/12 | 100/0/0 | 75/6/19 |
| SVD | 88/8/4 | 67/25/8 | 83/17/0 | 71/25/4 | 96/0/4 | 58/25/17 |
| AIE | 87/8/6 | 70/21/9 | 74/21/6 | 72/19/9 | 96/2/2 | 77/15/8 |
| PD | 93/5/2 | 79/16/5 | 84/16/0 | 78/19/3 | 91/5/3 | 74/16/10 |
| PDD | 84/16/0 | 42/32/26 | 79/16/5 | 58/26/16 | 79/21/0 | 68/26/5 |
| DLB | 78/22/0 | 44/22/33 | 94/0/6 | 56/39/6 | 89/6/6 | 78/6/17 |
| AD | 80/20/0 | 67/17/17 | 69/22/9 | 69/24/7 | 87/9/4 | 70/7/22 |
| MCI | 86/14/0 | 57/43/0 | 100/0/0 | 71/29/0 | 100/0/0 | 71/29/0 |
| SCD | 74/21/5 | 86/9/5 | 65/33/2 | 65/26/9 | 91/7/2 | 79/14/7 |
| HC | 95/5/0 | 100/0/0 | 100/0/0 | 89/11/0 | 100/0/0 | 79/21/0 |

Each cell shows the percentage values of No Apathy, Moderate Apathy and Severe Apathy. For example, based on self-report AMI behavioural score, bvFTD’s data is 86/9/5, which means bvFTD had 86% no apathy, 9% moderate apathy and 5% severe apathy. SR = Self-report. CG = Caregiver-report. See Figure 6 for visualisation.

## ****Modelling results****

### Stepwise multiple linear regression predicting ARD from five ACE subscores

| Model Summary on predicting ARD from five ACE subscores | | | | | | | | | | | | | | | | | | | | | | | |
| --- | --- | --- | --- | --- | --- | --- | --- | --- | --- | --- | --- | --- | --- | --- | --- | --- | --- | --- | --- | --- | --- | --- | --- |
| Model | | R | | R² | | Adjusted R² | | RMSE | | AIC | | BIC | | R² Change | | F Change | | df1 | | df2 | | p | |
| M₀ |  | 0.000 |  | 0.000 |  | 0.000 |  | 0.746 |  | 710.078 |  | 717.577 |  | 0.000 |  |  |  | 0 |  | 313 |  |  |  |
| M₁ |  | 0.303 |  | 0.092 |  | 0.089 |  | 0.712 |  | 681.942 |  | 693.190 |  | 0.092 |  | 31.428 |  | 1 |  | 312 |  | < .001 |  |
| M₂ |  | 0.329 |  | 0.108 |  | 0.103 |  | 0.707 |  | 678.109 |  | 693.106 |  | 0.017 |  | 5.832 |  | 1 |  | 311 |  | 0.016 |  |
|  | | | | | | | | | | | | | | | | | | | | | | | |

| ANOVA | | | | | | | | | | | | | |
| --- | --- | --- | --- | --- | --- | --- | --- | --- | --- | --- | --- | --- | --- |
| Model | |  | | Sum of Squares | | df | | Mean Square | | F | | p | |
| M₁ |  | Regression |  | 15.941 |  | 1 |  | 15.941 |  | 31.428 |  | < .001 |  |
|  |  | Residual |  | 158.253 |  | 312 |  | 0.507 |  |  |  |  |  |
|  |  | Total |  | 174.194 |  | 313 |  |  |  |  |  |  |  |
| M₂ |  | Regression |  | 18.854 |  | 2 |  | 9.427 |  | 18.873 |  | < .001 |  |
|  |  | Residual |  | 155.341 |  | 311 |  | 0.499 |  |  |  |  |  |
|  |  | Total |  | 174.194 |  | 313 |  |  |  |  |  |  |  |
|  | | | | | | | | | | | | | |
| Note.  The intercept model is omitted, as no meaningful information can be shown. | | | | | | | | | | | | | |

| Coefficients | | | | | | | | | | | | | |
| --- | --- | --- | --- | --- | --- | --- | --- | --- | --- | --- | --- | --- | --- |
| Model | |  | | Unstandardized | | Standard Error | | Standardized | | t | | p | |
| M₀ |  | (Intercept) |  | 0.375 |  | 0.042 |  |  |  | 8.905 |  | < .001 |  |
| M₁ |  | (Intercept) |  | 1.013 |  | 0.121 |  |  |  | 8.391 |  | < .001 |  |
|  |  | ACE Memory |  | -0.034 |  | 0.006 |  | -0.303 |  | -5.606 |  | < .001 |  |
| M₂ |  | (Intercept) |  | 1.096 |  | 0.125 |  |  |  | 8.794 |  | < .001 |  |
|  |  | ACE Memory |  | -0.022 |  | 0.008 |  | -0.198 |  | -2.886 |  | 0.004 |  |
|  |  | ACE Fluency |  | -0.033 |  | 0.014 |  | -0.166 |  | -2.415 |  | 0.016 |  |
|  | | | | | | | | | | | | | |
| Note.  The following covariates were considered but not included: ACE Attention, ACE Language, ACE Visuospatial. | | | | | | | | | | | | | |

| Collinearity Diagnostics | | | | | | | | | | | | | |
| --- | --- | --- | --- | --- | --- | --- | --- | --- | --- | --- | --- | --- | --- |
|  | | | | | | | | Variance Proportions | | | | | |
| Model | | Dimension | | Eigenvalue | | Condition Index | | (Intercept) | | ACE Memory | | ACE Fluency | |
| M₁ |  | 1 |  | 1.943 |  | 1.000 |  | 0.029 |  | 0.029 |  |  |  |
|  |  | 2 |  | 0.057 |  | 5.838 |  | 0.971 |  | 0.971 |  |  |  |
| M₂ |  | 1 |  | 2.880 |  | 1.000 |  | 0.012 |  | 0.008 |  | 0.010 |  |
|  |  | 2 |  | 0.075 |  | 6.181 |  | 0.798 |  | 0.016 |  | 0.457 |  |
|  |  | 3 |  | 0.044 |  | 8.052 |  | 0.191 |  | 0.976 |  | 0.533 |  |
|  | | | | | | | | | | | | | |
| Note.  The intercept model is omitted, as no meaningful information can be shown. | | | | | | | | | | | | | |

### Multiple linear regression predicting total AMI-CG from AMI-SR subscales and ACE

| Model Summary on predicting AMI-CG total from 3 AMI-SR subscores and 5 ACE subscores | | | | | | | | | | | | | | | | | | | | | | | |
| --- | --- | --- | --- | --- | --- | --- | --- | --- | --- | --- | --- | --- | --- | --- | --- | --- | --- | --- | --- | --- | --- | --- | --- |
| Model | | R | | R² | | Adjusted R² | | RMSE | | AIC | | BIC | | R² Change | | F Change | | df1 | | df2 | | p | |
| M₀ |  | 0.379 |  | 0.143 |  | 0.135 |  | 0.695 |  | 668.615 |  | 687.362 |  | 0.143 |  | 17.306 |  | 3 |  | 310 |  | < .001 |  |
| M₁ |  | 0.486 |  | 0.236 |  | 0.226 |  | 0.657 |  | 634.639 |  | 657.135 |  | 0.093 |  | 37.511 |  | 1 |  | 309 |  | < .001 |  |
| M₂ |  | 0.505 |  | 0.255 |  | 0.243 |  | 0.650 |  | 628.634 |  | 654.880 |  | 0.019 |  | 7.953 |  | 1 |  | 308 |  | 0.005 |  |
|  | | | | | | | | | | | | | | | | | | | | | | | |

| *ANOVA* | | | | | | | | | | | | | |
| --- | --- | --- | --- | --- | --- | --- | --- | --- | --- | --- | --- | --- | --- |
| Model | |  | | Sum of Squares | | df | | Mean Square | | F | | p | |
| M₀ |  | Regression |  | 25.081 |  | 3 |  | 8.360 |  | 17.306 |  | < .001 |  |
|  |  | Residual |  | 149.757 |  | 310 |  | 0.483 |  |  |  |  |  |
|  |  | Total |  | 174.838 |  | 313 |  |  |  |  |  |  |  |
| M₁ |  | Regression |  | 41.293 |  | 4 |  | 10.323 |  | 23.886 |  | < .001 |  |
|  |  | Residual |  | 133.545 |  | 309 |  | 0.432 |  |  |  |  |  |
|  |  | Total |  | 174.838 |  | 313 |  |  |  |  |  |  |  |
| M₂ |  | Regression |  | 44.654 |  | 5 |  | 8.931 |  | 21.129 |  | < .001 |  |
|  |  | Residual |  | 130.184 |  | 308 |  | 0.423 |  |  |  |  |  |
|  |  | Total |  | 174.838 |  | 313 |  |  |  |  |  |  |  |
|  | | | | | | | | | | | | | |

| *Coefficients* | | | | | | | | | | | | | |
| --- | --- | --- | --- | --- | --- | --- | --- | --- | --- | --- | --- | --- | --- |
| Model | |  | | Unstandardized | | Standard Error | | Standardized | | t | | p | |
| M₀ |  | (Intercept) |  | 1.094 |  | 0.110 |  |  |  | 9.979 |  | < .001 |  |
|  |  | ami_behaviour |  | 0.141 |  | 0.060 |  | 0.143 |  | 2.363 |  | 0.019 |  |
|  |  | ami_social |  | 0.164 |  | 0.057 |  | 0.184 |  | 2.864 |  | 0.004 |  |
|  |  | ami_emotional |  | 0.218 |  | 0.070 |  | 0.175 |  | 3.097 |  | 0.002 |  |
| M₁ |  | (Intercept) |  | 1.675 |  | 0.141 |  |  |  | 11.917 |  | < .001 |  |
|  |  | ami_behaviour |  | 0.146 |  | 0.056 |  | 0.149 |  | 2.590 |  | 0.010 |  |
|  |  | ami_social |  | 0.154 |  | 0.054 |  | 0.172 |  | 2.837 |  | 0.005 |  |
|  |  | ami_emotional |  | 0.203 |  | 0.067 |  | 0.163 |  | 3.051 |  | 0.002 |  |
|  |  | ace_fluency |  | -0.061 |  | 0.010 |  | -0.305 |  | -6.125 |  | < .001 |  |
| M₂ |  | (Intercept) |  | 1.838 |  | 0.151 |  |  |  | 12.211 |  | < .001 |  |
|  |  | ami_behaviour |  | 0.153 |  | 0.056 |  | 0.156 |  | 2.747 |  | 0.006 |  |
|  |  | ami_social |  | 0.155 |  | 0.054 |  | 0.174 |  | 2.895 |  | 0.004 |  |
|  |  | ami_emotional |  | 0.200 |  | 0.066 |  | 0.160 |  | 3.031 |  | 0.003 |  |
|  |  | ace_fluency |  | -0.039 |  | 0.013 |  | -0.193 |  | -3.055 |  | 0.002 |  |
|  |  | ace_memory |  | -0.020 |  | 0.007 |  | -0.178 |  | -2.820 |  | 0.005 |  |
|  | | | | | | | | | | | | | |
| Note.  The following covariates were considered but not included: ace_attention, ace_language, ace_visuospatial. | | | | | | | | | | | | | |

| *Collinearity Diagnostics* | | | | | | | | | | | | | | | | | | | |
| --- | --- | --- | --- | --- | --- | --- | --- | --- | --- | --- | --- | --- | --- | --- | --- | --- | --- | --- | --- |
|  | | | | | | | | Variance Proportions | | | | | | | | | | | |
| Model | | Dimension | | Eigenvalue | | Condition Index | | (Intercept) | | ami_behaviour | | ami_social | | ami_emotional | | ace_flu | | ace_mem | |
| M₀ |  | 1 |  | 3.622 |  | 1.000 |  | 0.009 |  | 0.010 |  | 0.009 |  | 0.015 |  |  |  |  |  |
|  |  | 2 |  | 0.197 |  | 4.288 |  | 0.008 |  | 0.199 |  | 0.018 |  | 0.791 |  |  |  |  |  |
|  |  | 3 |  | 0.095 |  | 6.163 |  | 0.976 |  | 0.149 |  | 0.186 |  | 0.064 |  |  |  |  |  |
|  |  | 4 |  | 0.086 |  | 6.494 |  | 0.007 |  | 0.642 |  | 0.787 |  | 0.130 |  |  |  |  |  |
| M₁ |  | 1 |  | 4.448 |  | 1.000 |  | 0.003 |  | 0.007 |  | 0.006 |  | 0.010 |  | 0.006 |  |  |  |
|  |  | 2 |  | 0.229 |  | 4.405 |  | 0.016 |  | 0.000 |  | 0.024 |  | 0.451 |  | 0.276 |  |  |  |
|  |  | 3 |  | 0.185 |  | 4.902 |  | 0.004 |  | 0.325 |  | 0.084 |  | 0.364 |  | 0.113 |  |  |  |
|  |  | 4 |  | 0.086 |  | 7.196 |  | 0.001 |  | 0.612 |  | 0.821 |  | 0.121 |  | 0.000 |  |  |  |
|  |  | 5 |  | 0.052 |  | 9.290 |  | 0.976 |  | 0.056 |  | 0.065 |  | 0.055 |  | 0.605 |  |  |  |
| M₂ |  | 1 |  | 5.343 |  | 1.000 |  | 0.002 |  | 0.004 |  | 0.004 |  | 0.006 |  | 0.003 |  | 0.002 |  |
|  |  | 2 |  | 0.286 |  | 4.325 |  | 0.003 |  | 0.027 |  | 0.051 |  | 0.253 |  | 0.088 |  | 0.047 |  |
|  |  | 3 |  | 0.190 |  | 5.310 |  | 0.000 |  | 0.305 |  | 0.060 |  | 0.561 |  | 0.012 |  | 0.004 |  |
|  |  | 4 |  | 0.086 |  | 7.887 |  | 0.000 |  | 0.611 |  | 0.822 |  | 0.120 |  | 0.000 |  | 0.000 |  |
|  |  | 5 |  | 0.053 |  | 10.056 |  | 0.532 |  | 0.044 |  | 0.048 |  | 0.036 |  | 0.664 |  | 0.098 |  |
|  |  | 6 |  | 0.043 |  | 11.195 |  | 0.463 |  | 0.009 |  | 0.016 |  | 0.023 |  | 0.234 |  | 0.849 |  |
|  | | | | | | | | | | | | | | | | | | | |
